# The role of nanotherapy in head and neck squamous cell carcinoma by targeting tumor microenvironment

**DOI:** 10.3389/fimmu.2023.1189323

**Published:** 2023-05-24

**Authors:** Ye Zhang, Pengbo Dong, Lu Yang

**Affiliations:** ^1^ Department of Radiation Oncology, Cancer Hospital of Dalian University of Technology/Liaoning Cancer Hospital and Institute, Shenyang, China; ^2^ School of Energy and Power Engineering, Dalian University of Technology, Dalian, China; ^3^ Department of Internal Medicine, Cancer Hospital of Dalian University of Technology/Liaoning Cancer Hospital and Institute, Shenyang, China

**Keywords:** nanotherapy, immune microenvironment, head and neck squamous cell carcinoma, tumor microenvironment, immune response

## Abstract

Head and neck squamous cell carcinomas (HNSCCs) refers to a group of highly malignant and pathogenically complex tumors. Traditional treatment methods include surgery, radiotherapy, and chemotherapy. However, with advancements in genetics, molecular medicine, and nanotherapy, more effective and safer treatments have been developed. Nanotherapy, in particular, has the potential to be an alternative therapeutic option for HNSCC patients, given its advantageous targeting capabilities, low toxicity and modifiability. Recent research has highlighted the important role of the tumor microenvironment (TME) in the development of HNSCC. The TME is composed of various cellular components, such as fibroblasts, vascular endothelial cells, and immune cells, as well as non-cellular agents such as cytokines, chemokines, growth factors, extracellular matrix (ECM), and extracellular vesicles (EVs). These components greatly influence the prognosis and therapeutic efficacy of HNSCC, making the TME a potential target for treatment using nanotherapy. By regulating angiogenesis, immune response, tumor metastasis and other factors, nanotherapy can potentially alleviate HNSCC symptoms. This review aims to summarize and discuss the application of nanotherapy that targets HNSCC’s TME. We highlight the therapeutic value of nanotherapy for HNSCC patients.

## Introduction

Head and neck squamous cell carcinoma (HNSCC) is a neoplastic disease that is prevalent worldwide and is on the rise (1). A vast majority-over 60% of HNSCC patients are diagnosed at III or IV tumor stages, and around 10% of patients have distant metastases (2). The consumption of alcohol and tobacco are recognized risk factors for HNSCC, however, some recent studies have revealed a strong association between human papillomavirus (HPV) infection and HNSCC (3). Currently, comprehensive therapy with the combination of chemotherapy, radiation, and surgery are available for HNSCC (4). Nonetheless, the 5-year survival rate of HNSCC patients remains unsatisfactory due to various reasons, including late stage detection, the likelihood of recurrence, severe side effects, and resistance to medication (5, 6).

**Figure 1:**
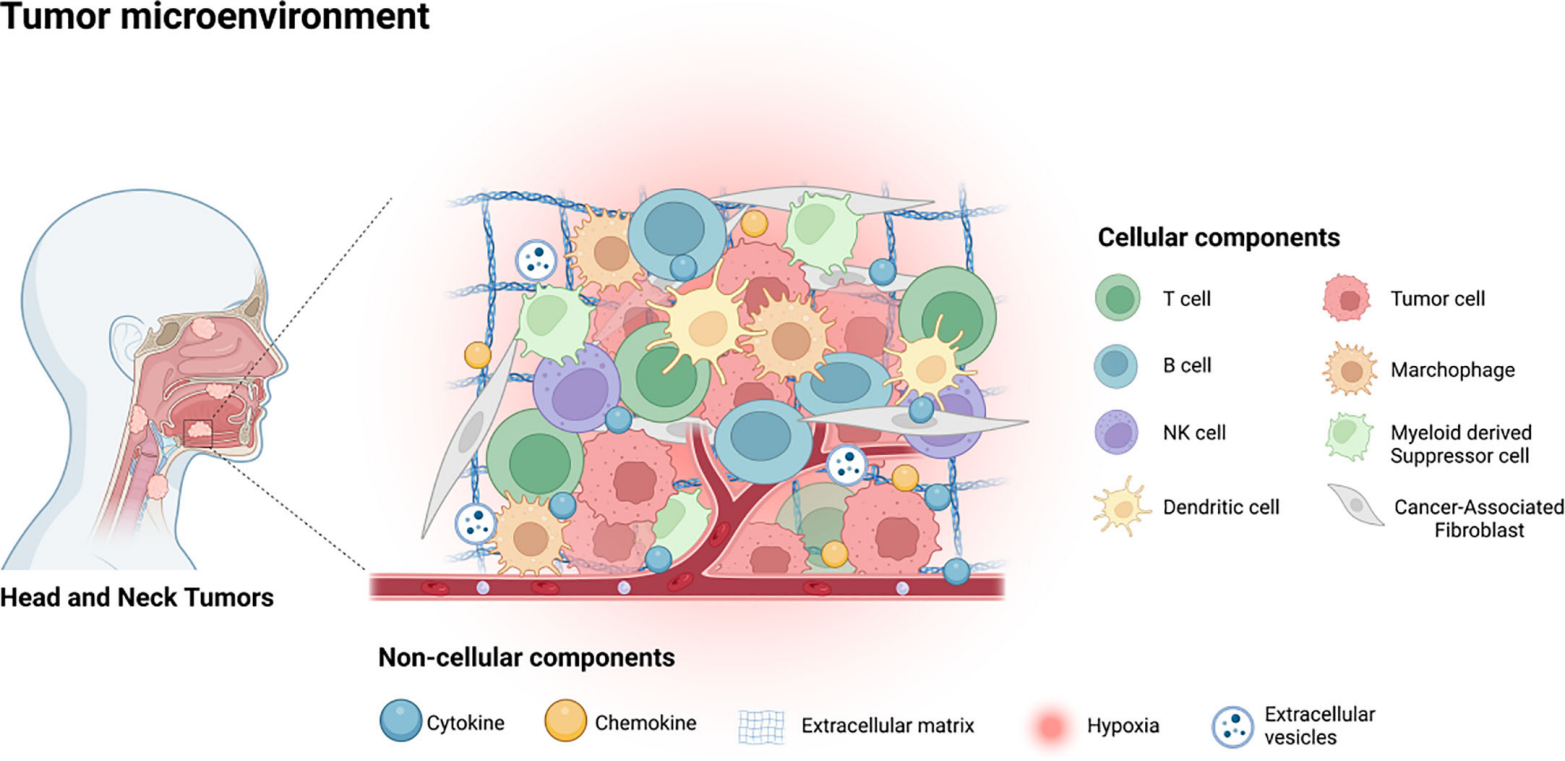
The tumor microenvironment of HNSCC contains various cellular components, including tumor cells, immune related cells, stromal cells and some non-cellular components, such as cytokines, chemokines, EVs, ECM and the hypoxia environment.

The interaction between tumor cells and their tumor microenvironment (TME) plays a pivotal role in the progression of malignancy and the poorer prognoses of patients, as has been well-documented (7). Therefore, the potential mechanism under the high rate of metastasis and recurrence of HNSCC is likely attributed to the crosstalk between the tumor cells and TME (8). The TME comprises various types of cells that include tumor associated fibroblasts, vascular endothelial cells (EC), adipocytes, mesenchymal stem cells (MSCs), and immune related cells, and diverse non-cellular components such as cytokines, chemokines, the extracellular matrix (ECM), and extracellular vesicles (EVs), as illustrated in **Figure 1** (7, 9, 10). As it is well-established that TME plays key roles in HNSCC progression and treatment resistance, targeting the constituents of TME for therapeutic benefits in HNSCC patients is gaining increased attention (11).

Nanotechnology has emerged as a promising area of research in various fields, including medicine and oncology, which has led to significant advancements in the diagnosis and treatment of cancer (12). Owing to the parallel expansion of biotechnology and nanomedicine, nanotherapy is perceived as a novel therapeutic approach for the management of different types of cancers (13). In particular, nanotherapy has exhibited immense potential for the treatment of head and neck squamous cell carcinoma (HNSCC) by augmenting the efficacy of radiotherapy, chemotherapy and immunotherapy through the incorporation of multiple drugs and/or molecules (12, 14). This review aims to provide a comprehensive overview of the current developments in nanotherapy targeting the tumor microenvironment (TME) in HNSCC, emphasizing the therapeutic benefits of these novel interventions.

## Nanotherapy in HNSCC

HNSCC therapeutic options traditionally comprise surgery, chemotherapy, radiotherapy, and combinations thereof. Surgical resection is particularly effective in treating carcinoma *in situ* or early-stage cancer, with chemotherapy presently regarded as the standard treatment scheme (15). However, surgery is not recommended for patients with late-stage or those with widely distant metastasis. Chemotherapy is also subject to limitations, including non-specific targeting, cytotoxicity, short half-life, poor solubility, drug resistance, and undesirable side effects (16). Consequently, the development of a drug delivery system capable of precisely targeting the tumor region is urgently needed. In recent years, nanotechnology has emerged as a promising area in medicine and oncology, with nanotherapy being extensively studied in cancer. Nanotherapy has several significant advantages, including accuracy, safety, modifiability, and biocompatibility, which may potentially address the limitations of conventional therapies (17, 18). Nanoparticles are defined as particles with nanometer size, superparamagnetic behavior, high surface-to-volume ratio, and unique fluorescence properties (19), Among the most widely researched nanoparticles in the medical field are liposomes, polymeric nanoparticles (PNPs), monoclonal antibody nanoparticles, metallic nanoparticles, among others, which can be employed for drug delivery and release (20, 21).

**Figure 2:**
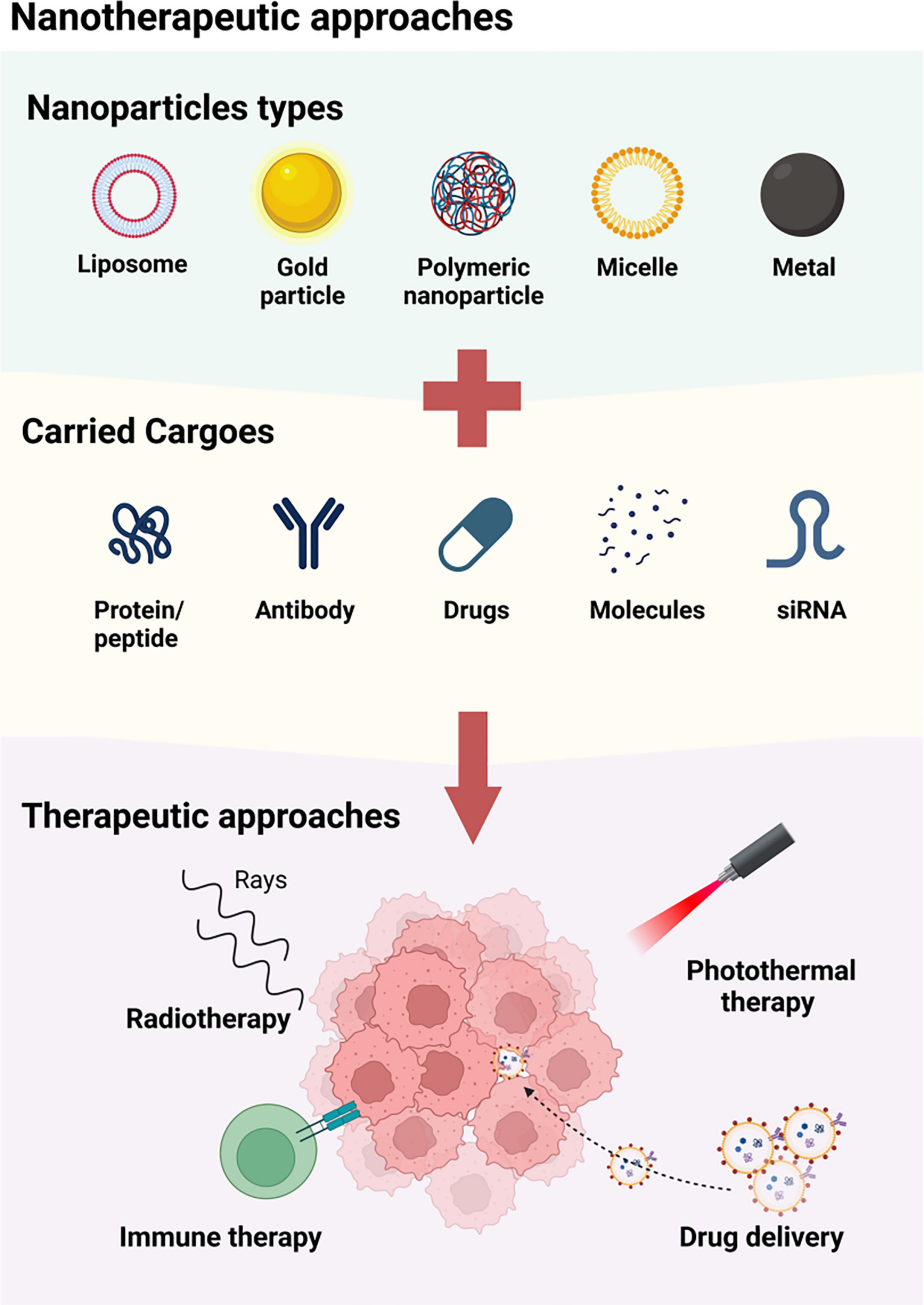
The available approaches of nanotherapy for HNSCC. A variety of nanoparticles, including liposome, metal particle, polymeric nanoparticle, micelle and magenic particles, are used for the treatment of HNSCC by carrying protein/peptide, antibodies, drugs, molecules and siRNA. These nanoparticles may function by enhancing the radiotherapy, combining photothermal therapy, inducing immune therapy, and accurately delivering agents to TME.

Recent years have seen numerous proposed nanotherapies for the treatment of head and neck squamous cell carcinoma (HNSCC) (**Figure 2**). Dihydroartemisinin (DHA) is one such therapy, but its poor solubility and short half-life in blood has limited its efficacy against HNSCC (22). To address these limitations, a magnetic dihydroartemisinin nano-liposome was designed and constructed to enhance the targeted delivery and bioavailability of DHA. The efficacy of this liposome was confirmed *via in vitro* and *in vivo* assays, demonstrating its potential to suppress tumor growth (23). Paclitaxel (PTX) is another chemotherapeutic drug widely used to treat locally advanced HNSCC (24), but its clinical use has been limited by severe side effects (25). To improve its therapeutic value, a polymeric nanodrug system was developed to target the transmission of PTX. This system displayed higher efficacy and fewer adverse reactions than free PTX in a HNSCC mouse xenograft model (26). Future research is required to provide more extensive evidence of the application of PTX-NPs in HNSCC patients. Additionally, zinc oxide-based (ZnO) nanoparticles (NPs) have been shown to have anti-tumor properties (27–29), with the ability to inhibit the viability of HNSCC cells (30). However, current studies have only been conducted at the cellular level, and further animal studies and clinical assays are necessary to fully determine the therapeutic value of ZnO-NPs for HNSCC patients.

## Nanotherapy targeting TME in HNSCC

**Figure 3:**
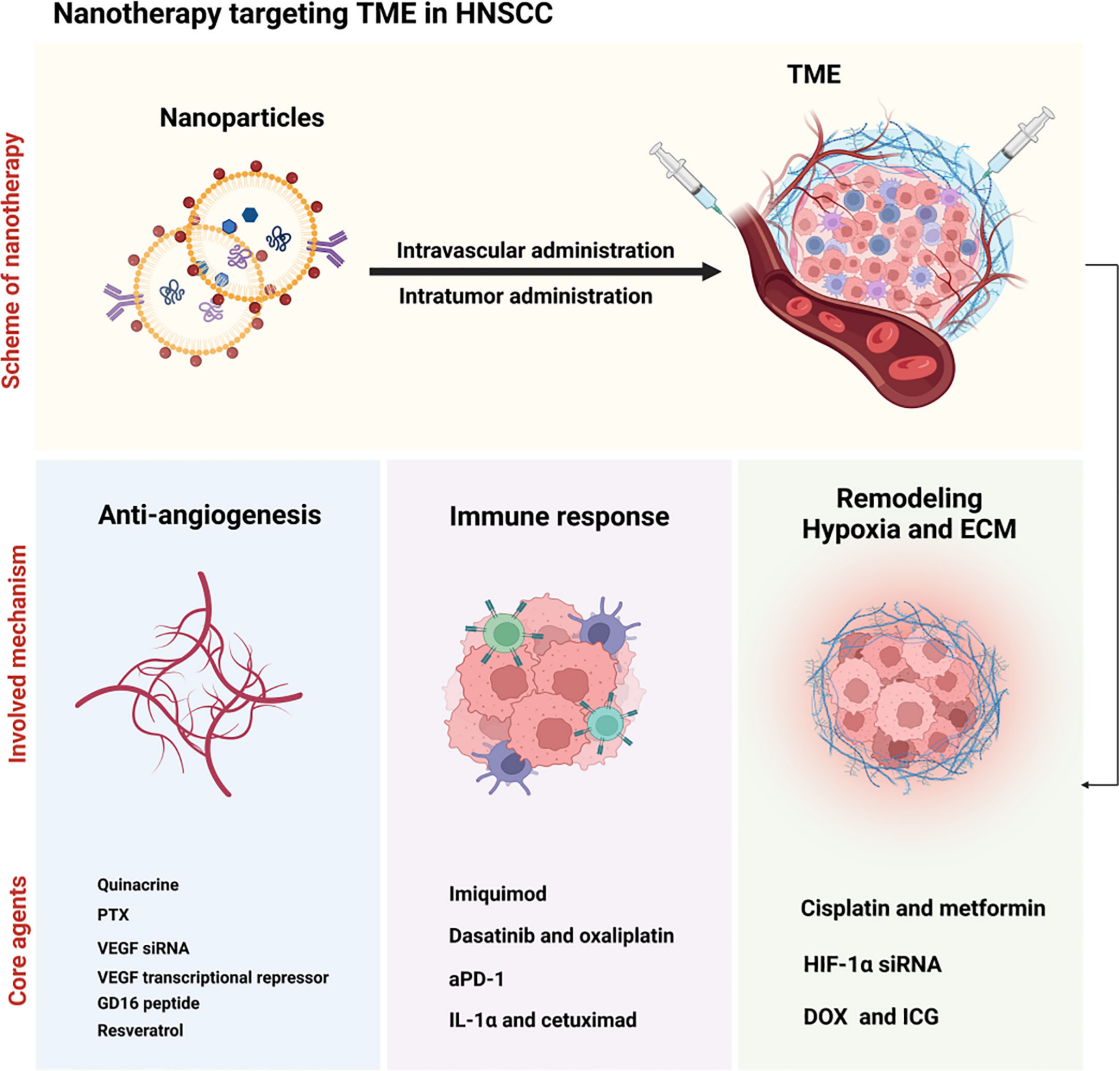
Illustration of nanotherapy targeting TME in HNSCC. Nanoparticles were injected intravascular or intratumor and subsequently induced the anti-angiogenesis (with quinacrine, PTX, VEGF siRNA, VEGF transcriptional repressor, GD16 peptide and resveratrol as the core agents), immune response (with imiquimod, dasatinib and oxaliplatin, aPD-1, and IL-1α and cetuximad as the critical agents), and the remodeling of hypoxia and ECM (with cisplatin and metformin, HIF-1α siRNA and DOX and ICG) in the TME.

Several nanotherapies have demonstrated effectiveness in enhancing the prognosis of tumor patients through targeting TME components, in addition to acting against tumor cells (31). Targeting TME presents significant therapeutic advantages compared to direct cancer cell targeting. This is due, in part, to the unstable genome of cancer cells, predisposing them to drug resistance, while TME-associated non-cancer cell genomes are generally more stable and susceptive (7). Consequently, an increasing number of studies have investigated the targeting of TME components in nanotherapies, including those designed for HNSCC treatment (see **Figure 3**) (32, 33).

## Nanotherapy antagonizes angiogenesis in HNSCC

Angiogenesis is a biological process that plays a crucial role in the formation of new blood vessels, enabling the delivery of oxygen and nutrients to all parts of the body. In the case of a tumor, angiogenesis is activated to support the rapid growth of cancer cells by developing an abundant vascular network in the tumor microenvironment (TME) (34). Recent evidence has shown that angiogenesis is a hallmark of aggressive cancers and plays a pivotal role in driving the malignant transition of tumors (35). Although anti-angiogenic drugs have been developed to prevent the formation of new blood vessels or break down existing vessels by targeting endothelial receptors and angiogenic-related cytokines, the clinical trials analyzing 38 studies found no significant benefit of angiogenesis inhibitors in patients with head and neck squamous cell carcinoma (HNSCC) (36, 37). Moreover, angiogenesis inhibitors were associated with unexpected toxicity. Nevertheless, nanoparticles loaded with angiogenesis inhibitors have shown great efficacy and safety in HNSCC patients (**Table 1**). A hybrid-nanoparticle (QAuNP) formulated with quinacrine and gold effectively attenuated angiogenesis, metastasis, and cancer stem cell proliferation in oral cancer (38). The therapeutic mechanism was associated with the modulation of the cytokine profile in the TME *via* the p53/p21 signaling pathway. The results suggest that nanotherapy could be a potential strategy to counteract angiogenesis in HNSCC. Paclitaxel (PTX) has been recognized as an effective chemotherapeutic agent for HNSCC, but its clinical utility is limited by side effects. To overcome this obstacle, a polymeric nanocarrier system (PTX-NPs) was developed for the delivery of PTX. In an HNSCC cancer model, PTX-NPs significantly inhibited tumor growth, possibly by regulating cell viability, angiogenesis, and oxidative stress. Notably, the expression of angiogenesis markers, such as Factor VIII, CD31, and CD34, was markedly reduced (26). Further research is warranted to evaluate the efficacy and safety of PTX-NPs in human subjects.

**Table 1:** Nanotherapies applied to antagonize angiogenesis in HNSCC.

Nanocarriers	Agent	Name	Mechanism of action	Ref
Polymer based nanoparticles	Paclitaxel	PTX-NPs	Decreasing angiogenesis markers (Factor VIII, CD31, and CD34)	(26)
Gold Nanoparticles	Quinacrine	QAuNP	Modulation the cytokine profile in TME *via* p53/p21 signaling pathway	(38)
Liposomes	VEGF siRNA	LCP-NPs	siRNA-mediated VEGF gene silencing	(39)
Gold Nanoparticles	hyperthermia	GNR	Improved endocytosis of oncolytic Ad, transgene expression, viral replication	(40)
Peptide conjugated nanoparticles	Paclitaxel	GD16-PTX-NP	Target the angiogenic marker Dll4	(41)
Polymer based nanoparticles	Resveratrol	Res-Nano	Decreased of angiogenic markers (MMPs, iNOS, VEGF-A, etc.)	(42)

The process of angiogenesis is intricately regulated by a plethora of stimulators and inhibitors that influence the viability of endothelial cells. Of particular significance is the irregular expression of vascular endothelial growth factor (VEGF), which plays a pivotal role in the formation of blood vessels within the tumor microenvironment (TME) (43). Targeting the VEGF pathway has been identified as a promising technique for treating a range of cancers, such as renal cell carcinoma (44), gastric cancer (45), liver cancer (46) and HNSCC (47). Nonetheless, the administration of anti-VEGF inhibitors can result in adverse effects, such as hypertension, proteinuria, and insufficient therapeutic responses (48). To surmount these issues, Lecaros (39) employed lipid-calcium-phosphate nanoparticles (LCP-NPs) to transport VEGF siRNA, and noted that photodynamic therapy (PDT) in combination with LCP-NPs-VEGF siRNA exhibited effective anti-tumor effects in HNSCC by impeding angiogenesis. Gold nanorod (GNR)-mediated plasmonic photothermal therapy can stimulate mild hyperthermia (40), thereby catalyzing the uptake and subsequent gene expression of oncolytic adenovirus (Ad) in HNSCC cells (49). Remarkably, the combination of oncolytic Ad expressing VEGF transcriptional repressor and GNR led to strong tumor repression in HNSCC.

The formation of vascular networks in the tumor microenvironment (TME) involves various factors, including but not limited to Vascular Endothelial Growth Factor (VEGF), Platelet-Derived Growth Factor (PDGF-B), Interleukin-8 (IL-8), Delta-like Ligand 4 (Dll4), and the Transforming Growth Factor (TGF-β) families (50, 51). Notably, Dll4 is highly expressed on the surface of tumor vascular endothelial cells as a ligand of the Notch receptor. Numerous investigations have reported that interventions in the Dll4-Notch signaling pathway lead to tumor growth inhibition (51–53). A recent study demonstrated that GD16-PTX-NP, a nanotherapy drug delivery system consisting of nanoparticles carrying paclitaxel conjugated with GD16 peptide, targeted Dll4 in human head and neck squamous cell carcinoma (HNSCC) FaDu xenograft mice. This nanodrug delivery system non-toxically and steadily released drugs, with a favorable long-circulating characteristic *in vivo*. Therefore, this study highlights the efficacy of Dll4-targeted nanodrug therapy in treating HNSCC (41).

Resveratrol has been widely investigated for its anti-tumor properties, specifically its anti-inflammatory, anti-metastatic and anti-angiogenic effects. However, its clinical use is restricted due to its short lifespan and poor pharmacokinetic profile (54). To address these limitations, Pradhan et al. (42) developed a nanoscale formulation of Resveratrol, termed Res-Nano, which exhibits promising results against metastasis and angiogenesis through the targeted modulation of tumor-associated macrophages in HNSCC. Nonetheless, further elucidation of the biochemical mechanisms mediating the effects of Res-Nano at the molecular level is necessary.

## Nanotherapy on improving blood vessel functions

There are currently several nanotherapies being tested in clinical trials as potential treatments for head and neck squamous cell carcinoma (NHSCC) (55–57). These nanotherapies use nanoparticles that can target cancer cells and deliver anti-cancer drugs directly to them. One type of nanotherapy being tested is called Abraxane, which uses albumin nanoparticles to deliver chemotherapy drug paclitaxel (58). Another type is BIND-014, which uses polymer nanoparticles to deliver chemotherapy drug docetaxel (57). These nanotherapies have shown promising results so far, with some patients experiencing complete remission or significant tumor reduction (56–58). The dosages used in these trials vary depending on the specific therapy being tested, but they are generally well-tolerated by patients with manageable side effects (55, 58). Overall, the use of nanotherapies in treating NHSCC shows great promise as a potential new approach to cancer treatment (55, 59).

## Nanotherapy induces immune response

The tumor microenvironment (TME) plays a crucial role in promoting the growth of tumor cells by inducing severe immunosuppression through the close interaction between immune and tumor cells, as evidenced by a growing number of studies (60, 61), Although immunotherapy has shown promising results in combating tumors, its clinical application is currently limited by the risk of eliciting destructive autoimmunity (62). However, the emergence of nanotechnology has provided a potential avenue for safer, more targeted and effective cancer immunotherapy, owing to its tunable biodistribution, excellent biocompatibility, immunogenicity, precise targeting, and controlled drug release properties (63). Hence, an increasing trend is observed to leverage nanotherapy as an approach to improve the immune response of tumors by targeting the TME in head and neck squamous cell carcinoma (HNSCC), as summarized in **Table 2** (69).

**Table 2:** Nanotherapies induces immune response in HNSCC.

Nanoparticles	Targeting	Response	Advantages	Ref
Nanohydrogel	TAMs	Switch of M2-to-M1 macrophage, and activation of T cells	Temperature-dependent *in situ* gelation.	(64)
iPDPA	T cells	Increasing memory T cells and boosting T-cell cytotoxicity	pH-responsive	(65)
PEG-PLGA	PD-1	Inhibition of T-cell PD-1 receptors	Improving biological distribution and bioactivity of ICBs	(66)
AuNCs	PD-1	Inhibition of T-cell PD-1 receptors	Preventing the recurrence of local tumor	(67)
Polyanhydride	T cells	Inducing a T cell-mediated immune response with increased CD8+ T cells	Alternative fo EGFR+ patients	(68)

## Immune cells

In the context of tumor microenvironment (TME), the pathogenesis of head and neck squamous cell carcinoma (HNSCC) is influenced by the composition and frequency of immune cells, including dendritic cells (DCs), T and B cells, natural killer (NK) cells, tumor-associated macrophages (TAM), and eosinophils. Manipulation of these immune cells provides a promising avenue for HNSCC treatment (74, 75). Monocytes recruited to the tumor site in TME undergo differentiation into TAM *via* diverse signaling pathways (76, 77). Macrophages exhibit M1 or M2 polarization and activation markers whereby M1 macrophages mainly induce an inflammatory response, while M2 macrophages exert an anti-inflammatory effect (78). TAMs are commonly recognized as M2 macrophages due to their involvement in the suppression of local immunity and promotion of tumor growth in TME. Remarkably, Wu et al. (64) developed a physiologically responsive nanocomposite hydrogel that undergoes temperature-dependent *in-situ* gelation and starts degradation in TME to promote the switch from M2-to-M1 macrophages. This action activates T cells and attenuates tumor growth and metastasis in HNSCC. Nevertheless, optimal dosing for this nanoparticle in large animal models and clinical trials remains an area of interest for further inquiry. Src, a proto-oncogene, has been found to be aberrantly activated in multiple malignant tumors, including HNSCC (79). Dasatinib, which is a specific inhibitor of Src, has synergism in combination with chemotherapy, as well as outstanding immunomodulatory effects in HNSCC models (80). Recently, a pH-responsive nanoparticle (PDO NP) was synthesized by incorporating a molecular agent comprised of dasatinib and oxaliplatin into the amphiphilic block copolymer iPDPA. The resulting PDO NP demonstrated anti-tumor efficacy by activating T cell anti-tumor immune response and promoting the generation of memory T cells as well as boosting T-cell cytotoxicity in HNSCC (65). Combining chemotherapy with TME-pH-responsive nanotherapy can offer an improved immunotherapeutic approach for HNSCC.

## Immune checkpoints

Immunotherapy has emerged as a critical therapeutic strategy for the treatment of tumors in recent years. Among the various immunotherapies, immune checkpoint blockade (ICB) is a monoclonal antibody-based therapy that blocks the interaction between immune checkpoints expressed on the surface of immune cells and their ligands present on tumor cells (81). The primary targets for ICB are cytotoxic T lymphocyte-associated antigen 4 (CTLA-4), programmed cell death 1 (PD-1), and programmed cell death 1 ligand 1 (PD-L1), which have received significant attention (82). In the tumor microenvironment (TME), the PD-1/PD-L1 axis is mainly responsible for the interactions between immune and tumor cells (83).

The biological distribution and bioactivity of immunotherapy antibodies, such as PD-1 targeting antibody (aPD-1), can be altered by their binding to nanoparticles (NPs). Liposomes, dendrimers, and polymeric NPs have been introduced as appropriate delivery systems for immunotherapy antibodies (84, 85). The poly (ethylene glycol)-b-poly- (lactide-co-glycolide) (PEG-PLGA) nanoparticle exhibits significant advantages over others. Badiee and colleagues (66) conjugated aPD-1 to PEG-PLGA NPs (aPD-1 NPs), and in a mouse model of head and neck squamous cell carcinoma (HNSCC), they demonstrated that aPD-1 NPs inhibited T-cell PD-1 receptors and attenuated HNSCC cell growth. However, further studies are required to evaluate the effectiveness of aPD-1 NPs in other solid tumor types. Gold nanocages (AuNCs) have shown therapeutic potential in a variety of malignant tumors due to their porous surface and hollow interiors, tunable localized surface plasmon resonance (LSPR) in the near-infrared (NIR) region, and outstanding biocompatibility (86). Recently, aPD-1@AuNCs, a nano drug delivery system that loaded aPD-1 into AuNCs, contributed to postoperative antitumor immunity, preventing the recurrence of local tumors in HNSCC (67, 87, 88). These findings suggest the potential use of aPD-1@AuNCs as a therapeutic option for postoperative HNSCC patients. Although epidermal growth factor receptor (EGFR) is highly expressed in HNSCC, the application of the EGFR inhibitor cetuximab did not improve the long-term survival outcomes of HNSCC patients. Therefore, the development of novel means to enhance the efficacy of cetuximab is crucial. A recent study showed that the novel IL-1α-loaded polyanhydride nanoparticles (IL-1α-NP) exhibited a synergistic effect when combined with cetuximab by inducing a T cell-mediated immune response, including an increase in CD8+ T cells (68). Although IL-1α-NP has the potential to be a viable immunotherapy for EGFR-bearing HNSCC patients, whether it can still be therapeutically useful in HNSCC patients with EGFR mutations requires further investigation.

## Nanotherapy improves hypoxia

The hypoxic microenvironment of the tumor microenvironment (TME) results from the combination of tumor cells’ high oxygen consumption rates and the malfunctioning of newly formed blood vessels (89, 90). This hypoxia environment can cause the heterogeneity and reprogramming of tumor cells and consequently promote the growth and metastasis of cancer (91, 92). Moreover, it has been established that hypoxia significantly enhances tumor cells’ tolerance to conventional therapies such as chemotherapy, radiotherapy, and photodynamic therapy (PDT) (93–95). Additionally, hypoxia interferes with the host’s autoimmune response leading to a reduction in the efficacy of tumor immunotherapy (96). Thus, regulating the TME’s hypoxic environment is crucial for preventing tumor migration, recurrence, and improving anti-tumor therapy efficacy. Hyperbaric oxygen inhalation is the main clinical method for the remission of hypoxia; however, the abnormal vascular structure in the TME significantly restricts oxygen diffusion rates and accumulation concentrations (97). Furthermore, hyperbaric oxygen poisoning is a non-negligible side effect. Fortunately, the development of nanotechnology has led to the creation of nanosystems that provide new approaches for targeting hypoxia precisely (98). The treatment of HNSCC involves the use of a chlorin e6 (Ce6) and polyethylene glycol diamine (PEG) based nano drug delivery system (termed CECMa NPs) (70). This system efficiently targets the hypoxic tumor region and co-delivers cisplatin and metformin, leading to enhanced tumor suppression with low toxicity. Another effective treatment for HNSCC is photodynamic therapy (PDT), which heavily relies on photosensitizers (PS) made from nanomaterials (99). Among these materials, titanium dioxide (TiO2) stands out for its high levels of compatibility with the human body and has been used as a drug delivery system for tumor treatment (100). A recent study developed a hypoxia-adaptive nanoparticle TiO2@Ru@siRNA by attaching a Ru complex and a siRNA that targets hypoxia inducible factor-1α (HIF-1α) to TiO2 NPs. Using this nanotherapeutic approach, PDT mediated by TiO2@Ru@siRNA induced lysosomal damage when the photocytotoxicity index (PI) was above 2000. Furthermore, HIF-1α siRNA was released to target the HNSCC hypoxic microenvironment that remodeled the immune microenvironment and suppressed tumor growth (71). Clinical implementation of this nanotherapy strategy could lead to the development of more effective and safer HNSCC treatments (**Table 3**).

**Table 3:** Nanotherapies targeting non-cellular components in HNSCC.

Nanoparticles	Cargoes	Target	Response	Application	Refs
CECMa	Cisplatin and metformin	Hypoxia	Targeting the hypoxic tumor region, and co-delivers cisplatin and metformin	Co-delivers cisplatin and metformin	(70)
TiO2	Ru complex and HIF-1α siRNA	Hypoxia	Silencc of HIF-1α	Combined with PDT	(71)
Nanohydrogel	nano DOX and nano ICG	ECM	Sustained release nano DOX and nano ICG with the presence of MMPs	Combined with 808nm NIR irradiation	(72)
Extracellular vesicle	MMP13	ECM	Inducing EMT of the recipient normoxic cell	Knocking down MMP13 in EVs	(73)

## Nanotherapy responses to ECM

ECM is a is a non-cellular component of the tumor microenvironment (TME) that acts as a scaffold in the tumor and is tightly involved in the acceleration of cancer malignancy (101, 102). Additionally, the degradation of ECM is an essential characteristic of progressing tumors (103). During this degradative progression, the pivotal enzymes are matrix metalloproteinases (MMPs) (104). Under normal conditions, MMPs are not activated; however, under the TME, their activities become enhanced. By degrading proteins of ECM, MMPs mainly contribute to the tumorigenesis and malignancy of cancers, including head and neck squamous cell carcinoma (HNSCC) (105, 106). To control the release of nanodrug delivery systems precisely, several smart nanoparticles responding to distinct stimuli have been manufactured (107). Recently, smart drug delivery systems responsive to MMPs have been developed and exhibited great potential for the diagnosis and treatment of solid tumors (108). A nano doxorubicin (DOX)-indocyanine green (ICG)-MMPs-responsive hydrogel (NDIMH) was synthesized, which could sustainably release nano DOX and nano ICG in the presence of MMPs (72). The use of NDIMH, in combination with 808 nm NIR irradiation, has demonstrated photosensitivity and antitumor effects in HNSCC cells and xenograft models. Thus, NDIMH combined with NIR irradiation could be a promising chemophototherapy option for the treatment of HNSCC. However, the optimal dose and duration of NDIMH require further exploration before clinical application. Extracellular vesicles (EVs) are nano-sized vesicles released by almost all cell types that participate in intercellular communication in the TME (109). Shan et al. (73), have shown that the hypoxic TME significantly increases the expression levels of MMP13 in tumor-derived EVs. This increase in MMP13 expression levels was found to be HIF-1α-dependent and induced EMT of the recipient normoxic cell, leading to tumor invasion. Therefore, tumor-derived EVs may act as messengers that mediate the interaction between normoxic and hypoxic cancer cells by delivering MMP13 and remodeling the TME of HNSCC. These findings provide a potential therapeutic strategy for HNSCC by downregulating the expression of MMP13 in these EVs.

## Limitations of nanotherapy in clinics and possible solutions for toxicity and non-specific targeting

Although nanotherapies have shown tremendous potential in preclinical studies in the context of head and neck squamous cell carcinoma (HNSCC), translating these results to the clinic is still challenging. Achieving selective and efficient targeting to the tumor site while limiting off-target effects and systemic toxicity remains a major hurdle in the clinical application of nanotherapies (110). Toxicity and non-specific targeting are the two primary concerns associated with the clinical application of nanotherapies in HNSCC. Toxicity arises mainly from the intrinsic properties of nanoparticles, which can induce immune reactions, inflammation, and unwanted interactions with blood components. On the other hand, non-specific targeting results in the accumulation of nanoparticles in non-targeted tissues, which can cause tissue damage, decreased efficacy, and off-target effects (111). To overcome these barriers, several strategies have been proposed to enhance the specificity and efficiency of nanotherapies in HNSCC clinics. One such strategy is to design nanoparticles that can selectively target HNSCC cells and avoid normal cells by exploiting various physiological and pathological properties of the HNSCC microenvironment. Active targeting approaches utilize various surface ligands, such as antibodies, peptides, and aptamers, to enhance nanoparticle accumulation in HNSCC cells through specific interactions with receptors or proteins that are overexpressed in HNSCC tissues. Several examples of these targeted nanotherapies are under preclinical development for HNSCC (112).

Another approach to improve the specificity and efficacy of nanotherapies is by optimizing nanoparticle parameters, such as size, surface charge, and surface modification. Nanoparticle size plays a crucial role in determining their behavior and selectivity *in vivo*. Generally, smaller nanoparticles can penetrate the HNSCC microenvironment more efficiently and accumulate in tumor tissues through EPR effects. In addition, optimizing surface modification, such as PEGylation, can enhance nanoparticle circulation time, reduce opsonization, and improve targeting efficiency without inducing significant host immune responses (113). Other potential strategies to enhance specificity and minimize toxicity include the development of smart nanoparticles that can respond to specific stimuli in HNSCC tissues, such as acidity, hypoxia, and redox reactions (114). Such stimuli-responsive nanotherapies can accumulate and deliver drugs selectively to HNSCC tissues, improving efficacy while reducing systemic toxicity. Finally, developing reliable methods of characterizing nanoparticle formulations *in vitro* and *in vivo* is also essential for advancing nanotherapies in HNSCC. The development of standards for characterization and quantification of nanoparticles to ensure the safe clinical translation of the nanotherapies is critical (115).

In conclusion, nanotherapies have great potential for treating HNSCC by improving blood vessel functions. However, several obstacles must be overcome to translate these therapeutic strategies from preclinical models to clinical practice. Strategies such as active targeting, optimization of nanoparticle size and surface modifications, and the development of smart nanoparticles can help address the limitations of nanotherapies in HNSCC, offering promising avenues for the treatment of HNSCC and other cancers.

## Vascular promotion effect or blood vessel normalization

One of the critical goals of nanoparticle-based treatments for HNSCC is to promote blood vessel normalization. The process of blood vessel normalization aims to restore the function of abnormal, leaky blood vessels that are generated by the tumor microenvironment. Blood vessel normalization helps in reducing the hypoxic and acidic environment within the tumor, and it improves the tumor delivery of important therapeutics, such as chemotherapy drugs, radiation, and immunotherapies (116). Nanoparticles can promote blood vessel normalization by improving vessel perfusion, decreasing interstitial fluid pressure, and improving the structure and function of endothelial cells. Moreover, nanoparticles can target the tumor vasculature by exploiting various physiological hallmarks of tumor vessels, such as enhanced permeability and retention effect (EPR), angiogenesis, and the overexpression of specific receptors on the tumor vessels. This targeting capability enables the selective accumulation of nanotherapeutics within the tumor tissue, which can lead to further benefits in blood vessel normalization (117).

A study by Chen et al. (118) investigated the role of a dual-targeting liposome co-loaded with docetaxel and sorafenib in HNSCC. The liposome was coated with cRGD, a peptide ligand that targets integrin αvβ3 receptors, and a HNSCC-specific monoclonal antibody. The study reported a significant reduction in tumor growth and induced blood vessel normalization in HNSCC xenograft models. Nanotherapy has been proposed as a promising approach to improving blood vessel functions in HNSCC by promoting vascular normalization, which can reduce tumor hypoxia and interstitial fluid pressure, thereby enhancing drug delivery and increasing treatment efficacy (119). Various types of nanocarriers, such as liposomes, polymeric nanoparticles, and gold nanoparticles, have been designed to target the tumor vasculature and deliver drugs specifically to the tumor site (120). Recent studies have demonstrated the effectiveness of nanotherapy in improving blood vessel functions in HNSCC. For example, a study by Li et al. (121) showed that the use of liposomal oxaliplatin combined with apatinib, a VEGFR2 inhibitor, resulted in significant tumor growth inhibition and vascular normalization *in vitro* and *in vivo*. Similarly, Wang et al. (122) reported that gold nanostars with surface-modified hyaluronic acid could selectively accumulate in HNSCC tumors and induce blood vessel normalization, which led to the enhanced efficacy of photothermal therapy. Moreover, some studies suggest that nanotherapy can also enhance the anti-tumor immune response by modulating the tumor microenvironment. For instance, Zheng et al. (123) developed a dual-function nanoplatform that can deliver programmed death-ligand 1 (PD-L1) antibodies and tumor necrosis factor-alpha (TNF-α) siRNA to the HNSCC tumor microenvironment. The results showed that the nanoplatform could promote blood vessel normalization and induce a robust anti-tumor immune response. In conclusion, nanotherapy has shown great potential in improving blood vessel functions in HNSCC, primarily by promoting vascular normalization and enhancing drug delivery. The use of innovative nanoplatforms that can target the tumor vasculature and modulate the tumor microenvironment may further increase treatment efficacy and improve patient outcomes.

## Conclusions and perspectives

In recent decades, the clinical treatment modalities for patients with head and neck squamous cell carcinoma (HNSCC) have largely relied on approaches that suppress the tumorigenic activities of cancerous cells. However, due to the high degree of tumor heterogeneity, aggressiveness, and potential for distant metastasis, conventional therapies such as radiotherapy and chemotherapy often yield limited clinical benefits (124). Despite the potential utility of immunotherapy, which stimulates anti-tumor immune responses, the objective response rate (ORR) remains unsatisfactory (125). Thus, it is imperative to explore strategies that minimize the toxic and side effects of traditional therapies while enhancing the efficacy of immunotherapy. Given the deeper understanding of the tumor microenvironment (TME) and the progress in nanomaterial technology, nanotherapy targeting the TME has garnered growing attention. Nanotherapeutic systems can be custom designed to exhibit specificity for unique TME characteristics, such as low pH, hypoxia, tumor-associated macrophages (TAMs), and increased matrix metalloproteinases (MMPs) expression (126–128). Additionally, nanoparticles with loading capabilities for specific drugs and targets (129, 130) can serve as drug carriers for chemotherapy drugs, cytotoxic agents, and immune checkpoint inhibitors (131, 132). Such nano drug delivery systems offer superior TME targeting, protect the efficacy of loaded agents from premature degradation, and increase the drug concentration at the TME site. Moreover, potential off-target effects, side effects, and tissue toxicities associated with traditional drug therapies can be minimized. These advantages make nanotherapy a promising new approach for treating tumors, including HNSCC. Furthermore, intravenous and intratumoral injection methods are the two main approaches for administering nanoparticles. Compared to deeper tumors, HNSCC is relatively accessible, which provides the opportunity for repeated intratumoral injections, thereby enhancing the therapeutic outcome of nanotherapy for HNSCC.

Despite the extensive research reported on nanotherapy for cancer, most studies conducted thus far have been cell and animal-based and may not fully reflect the effects of these nanodrugs on the human body. Additionally, research has shown that animal models, which are primarily carcinoma *in situ* models, possess better retention capacity (enhanced permeation and retention effect) compared to humans (133). Nevertheless, research should also focus on cancer metastasis, which is common in malignant tumors. Hence, a comprehensive evaluation of nanotherapy is needed, utilizing animal models, primate models, and patient-derived xenograft (PDX) models, since PDX models replicate human cancer characteristics and exhibit approximately 90% accuracy in reflecting drug response (134). Despite the large number of related research, currently, only a few nanodrugs, mainly liposomes and simple nanoparticles, are approved for clinical use (135–137), while nanocarriers with more complex structures and agents present significant challenges in clinical transformation due to some inherent limitations and unresolved problems. Among the most critical concerns are the potential toxicity and side effects of nanotherapy-related nanomaterials, which remain unresolved (138, 139). Nanoparticles can cross physiological barriers, threatening the safety of other organs due to their small size. Previous research has shown that nanoparticles can cause free radicals, inducing cell injury by attacking membranes, organelles, and DNA (140, 141). Additionally, the potential impact of nanoparticles on normal cells and tissues surrounding tumor cells must be considered. To overcome these challenges, further efforts should focus on developing nanomaterials that offer greater biocompatibility by improving nanomaterial traits, such as size, shape, chemical modifications, and surface charge. It is crucial to conduct clinical research to profile the metabolism and bioavailability of nanoparticles in the human body. Moreover, continuing to improve nanodrug targeting is still a worthy direction of exploration to prevent the abnormal distribution of nanoparticles in the human body. Finally, addressing the efficient retention of nanoparticles within tumor tissues and their delivery to draining lymph nodes are highly desirable research directions (142).

Despite the fact that some nanoparticles can accumulate in the tumor microenvironment (TME), the heterogeneous and complex TME poses challenges to effective drug delivery, leading to poor distribution of carried agents and limited efficacy of nanotherapy. Moreover, the variable characteristics of TME among different tumors indicate the need for individually tailored nanotherapies. Additionally, effective nanotherapy targeting the TME must consider the specific phenotypic changes of cancer cells or non-tumor cells in TME to avoid harmful effects on normal cells.

Despite the absence of approved nanotherapies for clinical practice in HNSCC (12), ongoing clinical trials evaluate the potential of nanotherapy in treating HNSCC. In a study by Weiss et al., 38 eligible subjects receiving a combination of nano-albumin-paclitaxel, carboplatin, and cetuximab achieved a response rate (RR) of 76.3% against locally advanced HNSCC, highlighting the promising clinical value of nanodrugs. Recently, a phase I study examined the safety of radioenhancer nanoparticles (NBTXR3) combined with intensity-modulated radiation therapy (IMRT) in elderly or debilitated HNSCC patients with locally advanced tumors who were not eligible for chemoradiation (143, 144). The intratumoral administration of NBTXR3 was feasible and displayed a safety profile, supporting its further evaluation at the recommended phase II dose (RP2D). Although few clinical trials have explored the value of nanotherapy specifically targeting the TME in HNSCC patients, its potential advantages create promising prospects for the development of therapeutic strategies for HNSCC

## Author contributions

Original draft preparation, allocation, supplementation and editing: YZ and LY. Revision: PD. All authors have read and agreed to the published version of the manuscript.
